# Working Elements in Interventions to Reduce Problematic Alcohol Use According to Older Adults: A Realist Evaluation

**DOI:** 10.1177/07334648241311457

**Published:** 2025-02-08

**Authors:** Fieke A. E. van den Bulck, Andrea D. Rozema, Dike van de Mheen, Rikste Knijff, Rob H. L. M. Bovens, Sarah E. Stutterheim, Rik Crutzen

**Affiliations:** 1 Tranzo Scientific Center for Care and Wellbeing, School of Social and Behavioral Sciences, 7899 Tilburg University, Tilburg, The Netherlands; 2 Positive Lifestyle Foundation, Tilburg, The Netherlands; 3 Department of Health Promotion, Care and Public Health Research Institute (CAPHRI), 5211 Maastricht University, Maastricht, The Netherlands

**Keywords:** alcohol, alcohol treatment, intervention, prevention, realist approach, qualitative methods

## Abstract

This study explored working elements (E) and mechanisms (M) in interventions within different contexts (C), contributing to the outcome (O) of reducing (problematic) alcohol use among older adults. Guided by a realist evaluation approach, interviews with older adults participating in interventions (*N* = 20) were conducted. In different contexts, several working elements were identified: (1) receiving information about alcohol and health (risks); (2) paying attention to abstinence; (3) being in contact with and receiving support from peers and relatives; (4) maintaining contact with a practitioner or peer/buddy who is always or flexibly available for communication; and (5) learning to manage drinking behavior, and one important mechanism was identified: motivation. From the older adults’ perspectives, there was a need to focus on social contact and support from peers and relatives, tracking and reflection, and motivation to reduce problematic alcohol use.

**What this paper adds**

• Older adults with problematic alcohol use need social support from peers and relatives.
• Motivation is an important mechanism in alcohol interventions, which can be fostered by motivational interviewing.
• Tracking and reflecting on alcohol use can offer insights into potential misconceptions about older adults’ drinking behavior, underlying motives, and environments. These insights can assist in focusing on individual or age-specific needs.

**Application of study findings**

• Future research might focus on the perspectives of the relatives of older adults who participate in interventions, to examine their willingness to be involved in interventions.

## Introduction

Problematic alcohol use among older adults is a growing and public health problem (Breslow et al., 2017; Kim et al., 2012; Roche & Kostadinov, 2019). Alcohol use is related to increased morbidity and mortality and plays a role in, among other problems, falls, dementia, and psychological problems including depression among older adults (Coulson et al., 2014; Shield et al., 2016; Sorock et al., 2006; Thomas & Rockwood, 2001). These alcohol-related problems, combined with the aging of the world population, highlight the importance of intervening in problematic alcohol use among older adults (United Nations, 2021). In order to reduce problematic alcohol use among older adults, age-specific interventions are required (Wadd et al., 2011), since older adults tend to respond well to age-specific interventions (Blow et al., 2000).

Recently, a systematic review focused on understanding how (working elements (E)), in what context (C), and why (mechanisms (M)) interventions are successful in reducing (problematic) alcohol use among adults, and specifically older adults (O) (Boumans et al., 2022). Only three of the 61 included studies evaluated interventions for older adults specifically (Ettner et al., 2014; Fink et al., 2005; Kuerbis et al., 2015). Overall, three major working elements of interventions were identified, of which only the first two were found in interventions specifically for older adults: (1) providing information on the consequences of alcohol use; (2) personalized feedback about drinking behavior; and (3) being in contact with others and communicating with them about (alcohol) problems. Also, working elements were identified in six different contexts. First, interventions in the context with practitioner involvement delivered in person to individuals (C) highlighted attention to drinking behavior (E) and client–practitioner relationships (E). Second, interventions with practitioners delivered not in person to individuals (C) emphasized personal contact and feedback (E), and online communication (E). For interventions involving practitioners in person with relatives (C), the relationship status (E) and teaching the partner to deal with the drinking behavior of the client (E) were key. Group interventions with in-person practitioner involvement (C), particularly in workplace settings, focused on lifestyle motivation (E). Individual interventions without practitioner involvement (C), such as web-based and telephone-based methods (E), were effective. Finally, group interventions without in-person practitioner involvement (C) focused on abstinence (E).

This review showed that little is known about what exactly works for older people (Boumans et al., 2022). Two other reviews focused on the interventions’ efficacy for older adults (Armstrong-Moore et al., 2018; Kelly et al., 2018); however, clear descriptions of the included interventions were lacking, and it was therefore difficult to determine the working elements. Most studies investigating the mechanisms explaining why interventions lead to certain outcomes were not conducted among older adults (Mutschler et al., 2021; Newton et al., 2018) or are focusing on substance use and not solely on alcohol use (Shams et al., 2021). Two previous qualitative studies explored the preferences of older adults concerning substance use interventions. Older adults in treatment for substance use have preferred interventions which are (1) accessible, (2) led by warm, caring, and non-confrontational therapists, (3) able to provide peer support and individual attention, and (4) open to friend and family involvement (Holland et al., 2016). Some older adults preferred group interventions that were not focused solely on drinking, for example, relaxation and arts classes, because these group sessions helped fill their day and expanded their social circle (Haighton et al., 2016). Although there is information regarding older adults’ preferences, their perspectives on the working mechanisms of interventions remain unknown, which are needed for tailoring interventions to their unique age-specific needs.

The objective of this study was thus to explore the working elements (E) and mechanisms (M) in interventions within different contexts (C), contributing to the outcome (O) of reducing (problematic) alcohol use among older adults, from the perspective of older adults participating in interventions.

To achieve this objective, this study built on the aforementioned systematic review (Boumans et al., 2022) and used a realist evaluation (RE) approach. RE examines how interventions and their elements (E) work differently across contexts (C), because the mechanisms (M) needed for successful intervention outcomes (O) are activated to a different extent. Context refers to the setting in which an intervention takes place, and mechanism refers to the process triggered when people interact with elements within a certain context, leading to an effect or change. Thus, the interaction between elements, contexts, and mechanisms are crucial in determining the outcome of an intervention. RE unpacks the relationships between these constructs, which is called the context–element–mechanism–outcome (CEMO) configuration (Pawson et al., 1997). RE starts with formulating an initial program theory (IPT) based on CEMOs found in the literature. The review’s findings per the six different intervention contexts (Boumans et al., 2022) formed our IPT and were tested in this study with older adults to confirm, refute, or refine the CEMOs of the IPT.

## Method

### Study Design

Semi-structured interviews with an RE approach were conducted with older adults who participated in interventions to reduce their (problematic) alcohol use. This study was part of a larger project, investigating the perspectives of professionals (Van Den Bulck et al., 2024) and close relatives of older adults. Ethical approval was received for this study by the Ethics Review Board at Tilburg University (RP508). The COREQ guidelines were followed in reporting (Tong et al., 2007) (see Supplemental Material 2). All included participants read an informed consent form and gave oral informed consent prior to the interviews.

### Sampling and Recruitment

In this study, we included interventions if they (1) focused on preventing or reducing (problematic) alcohol use; (2) were provided in an individual and/or group setting with or without relative involvement; (3) were provided face to face, online, and/or via telephone; (4) were provided in one country because the functioning of interventions could be influenced by national healthcare policies, or cultural and social factors; and (5) were provided within the last year. Interventions were excluded if they (1) were not primarily aimed at lifestyle change; (2) were provided during admission to a clinic; (3) were group-specific interventions (e.g., for veterans or athletes); (4) focused on general health and lifestyle improvements without explicit mention of alcohol use; or (5) focused solely on recovery. Our primary focus was on elements contributing, reducing, or preventing problematic alcohol use. Given that recovery has a different and broader focus, their elements require separate consideration.

First, to find relevant interventions for older adults, we searched in the Dutch National Database Centre for Healthy Living (https://www.loketgezondleven.nl) that describes interventions. Additionally, we consulted the national working groups Elderly in the Dutch Partnership for Early Detection of Alcohol Problems, and Alcohol and Elderly of the Dutch Addiction Association. To identify additional interventions, we contacted professionals working with interventions in our network, posted messages on LinkedIn and made appeals in online support groups. Characteristics of the included interventions are shown in Supplemental Table 1.

Second, to recruit older adults, we approached professionals involved in the identified interventions by sending them an information letter, asking if they were willing to help with the recruitment. Professionals providing the interventions with practitioner involvement participating in interventions connected us to the older adults. We preferred recruiting older adults who had completed their participation. However, we also included some older adults during active participation. This method was less time-consuming for the practitioners and therefore the most effective for recruitment compared to approaching older adults no longer in contact with practitioners. Professionals who provided anonymous interventions could not assist us due to anonymity concerns, and therefore, no older adults from these interventions were included. FVDB also attended the Vitality Days to recruit older adults from that intervention. In the case of interventions without practitioner involvement, we recruit the older adults via calls in online support groups, and we directly approached older adults who previously shared posts about their experiences with interventions in these online support groups. Professionals working with NoThanks placed a call in their online newsletter to recruit older adults from that specific intervention.

Only older adults aged 55 years or older were included. Older adults who were interested received information about the study via email or by telephone. Subsequently, they were invited for an interview.

We contacted 38 older adults with an invitation to participate in an interview. Of those 38, 13 did not respond to our emails or calls, or were not interested in participating. Another five were excluded, one of them due to a failed audio recording and the other four due to their age and exclusion of interventions. In total, 20 older adults participated. The mean age of participants was 67.0 (*SD* = 5.2) years, 10 men and 10 women; and 19 participants were born in the Netherlands. Additional participant demographic characteristics are shown in Table 1.

**Table 1.** Participants’ Demographic Characteristics.

Baseline characteristic	Participants
	*n* = 20
Age (years)	
60–64	7
65–69	9
70–74	2
75–80	2
Marital status	
Married or registered partnership	11
Divorced	4
Widow/widower	4
Never been married	1
Education level^a^	
Lower education	3
Intermediate education	6
Higher education	11
Employment status	
Paid work (fulltime, parttime, and/or self-employed)	6
Volunteer work	6
Intervention^b^	
AA	4
Apps (Alcohol Freedom, Easyquit, Selfhelp Alcohol)	2
CBT for substance use	2
Fresh Onward	2
Moti-55	3
NoThanks	3
Online support group	2
Schema therapy	1
SoberCare	3
Vitality Days	2

^a^Lower education = primary education; the first 3 years of senior general secondary education (HAVO) and pre-university secondary education (VWO); prevocational secondary education (VMBO); lower secondary vocational training and assistant’s training (MBO-1), intermediate education = upper secondary education (HAVO/VWO), basic vocational training (MBO-2), vocational training (MBO-3), and middle management and specialist education (MBO-4), higher education = higher vocational education (HBO), university education (WO).

^b^Some participants participated in more than one intervention.

### Data Collection

Based on the participants’ preferences, interviews were conducted face to face (10%), online (25%), or by telephone (65%). Interviews were performed between June 2022 and August 2023, each lasting an average of 55.2 (range: 30.7–75.1) minutes. We used an interview guide (see Supplemental Material 1) that measured, first inductively, the perceived outcomes of the interventions (O), and what working elements (E) and mechanisms (M) contributed to these outcomes, and the influence of the contexts (C) in which the interventions were provided. At the end of the interview guide, we presented the previously found CEMO configurations of the IPT of the review of Boumans et al. (2022); we invited participants to confirm, refute, or refine the CEMOs.

### Data Analysis

Professional transcriptionists transcribed the interviews verbatim. FVDB drafted a code tree based on the IPT that encompassed the CEMO configurations. The IPT consisted of six combinations of contexts: (1) whether the client was in contact with a practitioner; (2) whether the intervention was provided in-person; and (3) regarding individual treatment, group treatment, or treatment with relatives’ involvement. However, in the current study, two contexts were added in the program theory since we collected data in two contexts beyond the IPT: no practitioner—in-person—group component and practitioner—not in-person—group component.

Independently and simultaneously, FVDB and RK coded three transcripts, seeking links between contexts, elements, mechanisms, and outcomes within the data (Pawson et al., 1997). After coding the first transcript, FVDB cross-checked RK’s configurations and codes. FVDB and RK iteratively discussed any differences in configurations or coding and made refinements where applicable. These steps were repeated for all three interviews until coding was fully aligned. Thereafter, FVDB independently coded the remaining interviews. When uncertainty arose regarding coding for specific data sections, FVDB discussed it with FK and ADR. To improve the dependability of the coding further, RC, RK, DVDM, RB, and ADR reviewed the codes and CEMOs iteratively and provided feedback. After discussing and receiving feedback, final adjustments were made by FVDB. Additionally, interpretations of data and how they related to the IPT were discussed to support the credibility of the findings. The data were analyzed using Atlas.ti in version 23.

## Results

The results were categorized according to the context in which the interventions were delivered. Table 2 provides the program theory with an overview of the key working elements (E), mechanisms (M), and outcomes (O). When these working elements were linked to specific interventions by the participants, the specific target group of this intervention was also indicated. Table 3 provides an overview of the summarized findings of the program theory.

**Table 2.** The Program Theory.

Context	Element of intervention	Mechanism		Outcome	Target group^b^	IPT^c^
A. Practitioner—in-person—individual	1. *Paying attention to drinking behavior*					✓
	1.1. Conversation with practitioner about drinking behavior	Thinking about own alcohol use	Awareness of excessive drinking	Less or no alcohol use	I, X	+
	1.2. Tracking alcohol use			Less alcohol use	II, X	+
	1.3. Learning to manage craving and drinking behavior			Less or no alcohol use	II, IV, X	+
	1.4. Receiving information about alcohol and health	a. Confirmation or awareness of health consequences		Less or no alcohol use	I, II, X	+
		b. Thinking about own alcohol use		Less or no alcohol use		
	1.5. Practitioner asks questions and reflects on alcohol use with client			Less alcohol use	I, II, IV, X	+
	2. *Communication approaches*					
	2.1. Receiving personalized content and conversation based on individual preferences and needs			No alcohol use	X	+
	3. *The relationship between the client and practitioner*					
	3.1. Trust, connection, and not being judged in contact with practitioner			Less alcohol use	I, II, IV, X	+
	3.2. Empathic behavior of practitioner	Client and practitioner collaborate in the identification of additional help, and their relationship improves		Less or no alcohol use	X	✓
	3.3. Critical and confronting behavior of practitioner	Insights and realization of the problem and a need to change			I, IV, X	+
	3.4. Practitioner listens				II, X	+
	3.5. Practitioner is available and contact is regular	Feelings of reliability and commitment			II, IV	+
	3.6. Practitioner is peer support worker	Recognition and connection in contact with practitioner			X	+
	4. *Participation setting*					
	4.1. Anonymous participation for environment				II, X	+
	5. Additional activities					
	5.1. Participating in self-help group			No alcohol use	I, IV	+
B. Practitioner—not in-person—individual	1. *Paying attention to drinking behavior*					
	1.1. Daily breath-testing with monitoring	Preventing the resumption of drinking		No alcohol use	IV	+
	1.2. Experiencing abstinence and health benefits	Motivation		No alcohol use	IV	+
	2. *Communication approaches*					
	2.1. Using the 12-step Minnesota model	Understanding the process of change		No alcohol use	IV	+
	2.2. Receiving personalized content based on individual preferences and needs				IV, X	+
	2.3. Reflecting with practitioner			No alcohol use	IV	+
	2.4. Receiving feedback				X	+
	^a^2.5. Online communication and feedback			Less or no alcohol use		∼
	^a^2.6. Personal contact and feedback			Less or no alcohol use		∼
	3. *The relationship between the client and practitioner*					
	3.1. Frequent and flexible contact with practitioner			No alcohol use	IV	+
	4. *Involving the relative in treatment*					
	4.1. Relative writes an impact letter	Being confronted with consequences of use			IV	+
	4.2. Partner monitors abstinence at home	Preventing the resumption of drinking			IV	+
	5. *Participation setting*					
	5.1. Online participation	No commute time			IV	+
C. Practitioner—in-person—relatives	1. The partner is taught to deal with drinking behavior	More understanding and support from partner for client	Motivation to abstain	Less or no alcohol use	X	✓
	2. The partner is taught to approach client positively			Less alcohol use	X	+
	3. Partner receives information about intervention	Partner can better support client			X	+
	4. Collaboration between client and relatives			Less or no alcohol use	I, X	+
	5. Receiving support of partner				X	+
D. Practitioner—in-person—group component	1. *Paying attention to drinking behavior*					
	1.1. Learning to manage craving and drinking behavior				IV, X	+
	1.2. The risks of drinking behavior are pointed out				IV	+
	1.3. Reflecting on cause of alcohol use with practitioner				IV	+
	2. *Paying attention to lifestyle*					
	2.1. Attention for lifestyle and motivating lifestyle change			Less or no alcohol use	X	+
	^a^2.2. Motivating to change lifestyle					∼
	3. *Communication approaches*					
	3.1. Receiving personalized content and conversation				IV, X	+
	3.2. Receiving feedback				IV, X	+
	4. *The relationship between the client and practitioner*					
	4.1. Practitioner is peer support worker	Feeling understood			IV, X	+
	5. *The relationship between the client and peers*					
	5.1. Contact, support, openness, and mutual respect	a. Enhancement of motivation to succeed		Less or no alcohol use	IV, X	+
		b. Feeling understood				
	5.2. Discussing choices, experiences, and emotions	a. Feeling supported by hearing others’ experiences		Less or no alcohol use	IV, X	+
		b. Learning from each other				
	5.3. Sharing negative experiences and struggles				IV, X	+
	5.4. Presence of peers with similar level of (problematic) alcohol use				IV, X	+
	6. *Participation setting*					
	6.1. Anonymous participation for environment				X	+
	^a^6.2. At work				X	-
	7. *Additional contact and activities*					
	7.1. Individual contact with practitioner	Receiving more personal attention			IV, X	+
	7.2. Activity engagement				IV	+
E. No practitioner—not in-person—individual	1. *Paying attention to drinking behavior*					
	1.1. Tracking alcohol use				I, X	+
	1.2. Daily abstinence goal	a. Small goals make abstinence more manageable			I, X	+
		b. Enhances a motivational mindset				
	1.3. The risks of drinking behavior are pointed out	Perspective regarding alcohol use changes			III	+
	1.4. Learning to manage craving and drinking behavior				III	+
	1.5. Participating in a temporary abstinence challenge	Motivation to abstain for a longer period			III, X	+
	2. *Participation setting*					
	2.1. Online, location, and time independent of participation	Low-threshold participation		Less or no alcohol use	I, III, X	+
	2.2. Receiving emails or newsletters regularly	Frequency strengthens or changes the perception of alcohol use			III	+
	^a^2.3. Web-based interventions					∼
	^a^2.4. Telephone-based interventions	Insight into use, which leads to realization of alcohol use				∼
	3. *Additional contact*					
	3.1. Being in contact with peer/buddy, relatives				III, X	+
F. No practitioner—not in-person—group component	1. *Paying attention to drinking behavior*					
	1.1. Focusing on abstinence			Less or no alcohol use	X	✓
	2. *The relationship between the client and peers*					
	2.1. Someone leading the group who monitors groups dynamic and content				X	+
	2.2. Sharing experiences and tips	Motivation to quit drinking			I, IV	+
	2.3. Presence of peers with similar level of (problematic) alcohol use				X	+
	3. *Additional contact*					
	3.1. Face to face meeting with peers				I,	+
	3.2. Contact with practitioner	Addressing problems more decisively			X	+
G. No practitioner—in-person—group component	1. *Paying attention to drinking behavior*					
	1.1. Attention for acknowledging problematic use				I,	+
	2. *Paying attention to lifestyle*					
	2.1. Sharing tips for activity engagement, or engaging in them together with group peers				I, X	+
	3. *Communication approaches*					
	3.1. Using the 12-step Minnesota model			Remain abstinent	I	+
	4. *The relationship between the client and peers*					
	4.1. Sharing experiences	Hearing about it and discussing it strengthens the realization of the need for change			I	+
	4.2. Peers are supportive and understanding	Feeling welcomed and connection			I	+
	4.3. Being able to reach each other at all times	Receiving help in difficult situations		No alcohol use	I	+
	5. *Participation setting*					
	5.1. Anonymous participation	Feeling secure			I	+
	5.2. Participating in sessions actively and regularly			Remain abstinent	I	+
	*6. Additional activities*					
	6.1. Engaging in social activities				I	+
	6.2. Entering treatment, in addition to attending a self-help group				I, X	+
H. Practitioner—not in-person—group component	1. *The relationship between the client and peers*					
	1.1. Participating in introduction week with peers	Group cohesion			IV	+
	1.2. Discussing difficulties				IV	+
	1.3. Peers have similar social status and functioning	Connection			IV	+
	2. *Participation setting*					
	2.1. Online, digital participation	No commute time			IV	+

^a^Elements of the Initial program theory that were refined, or not confirmed.

^b^Target group of the intervention. I = people with all types of alcohol use patterns; II = people with early-stage problematic alcohol use; III = people with all types of alcohol use patterns, except for (heavy) problematic alcohol use or alcohol addiction; IV = people with problematic alcohol use and alcohol addiction; X = no target group mentioned because these elements were derived from a CEMO configuration that we presented as statements during the interviews, or were not related to the intervention in which they participated.

^c^✓ = initial program theory confirmed; ∼ = initial program theory refined; + = program theory added; − = initial program theory not confirmed.

**Table 3.** Summary of the Program Theory.

Context	Element of intervention	Mechanism	Outcome
A. Practitioner—in-person—individual	Paying attention to drinking behavior	Changing perceptions on own use (1.1., 1.4.b.) and consequences (1.4.a.)	Less or no alcohol use
	Communication approaches		
	The relationship between the client and practitioner	Improving communication with practitioner (3.2.), changing perceptions on own use (3.3.), and social and emotional support (3.6) and feeling of reliability and commitment (3.5.)	
	Participation setting		
	Additional activities		
B. Practitioner—not in-person—individual	Paying attention to drinking behavior	Preventing the resumption of drinking (1.1.)	Less or no alcohol use
		Motivation (1.2.)	
	Communication approaches	Understanding of process of change (2.1.)	
	The relationship between the client and practitioner		
	Involving the relative in treatment	Changing perceptions on consequences (4.1.) and preventing the resumption of drinking (4.2.)	
	Participation setting	No commute time (5.1.)	
C. Practitioner—in-person—relatives	The partner is taught to deal with drinking behavior	More understanding and support from partner for client (1.) and motivation (1.)	Less or no alcohol use
	The partner is taught to approach client positively		
	Partner receives information about intervention	Support from partner for client (3)	
	Collaboration between client and relatives		
	Receiving support of partner		
D. Practitioner—in-person—group component	Paying attention to drinking behavior		Less or no alcohol use
	Paying attention to lifestyle		
	Communication approaches		
	The relationship between the client and practitioner	Emotional support (4.1.)	
	The relationship between the client and peers	Motivation (5.1.a.) and emotional and social support (5.1.b., 5.2.a., 5.2.b)	
	Participation setting		
	Additional contact and activities	Tailoring (7.1.)	
E. No practitioner—not in-person—individual	Paying attention to drinking behavior	Motivation (1.2.b., 1.5.), changing perceptions on use (1.3.), and making abstinence easier (1.2.a)	Less or no alcohol use
	Participation setting	Low-threshold participation (2.1.), and changing perceptions on use (2.2.)	
	Additional contact		
F. No practitioner—not in-person—group component	Paying attention to drinking behavior		Less or no alcohol use
	The relationship between the client and peers	Motivation (2.2).	
	Additional contact	Addressing problems more decisively (3.2.)	
G. No practitioner—in-person—group component	Paying attention to drinking behavior		Remain abstinent, no alcohol use
	Paying attention to lifestyle		
	Communication approaches		
	The relationship between the client and peers	Changing perceptions on use (4.1.)	
		Feeling welcomed and connection (4.2.)	
		Social support (4.3.)	
	Participation setting	Feeling secure (5.1.)	
	Additional activities		
H. Practitioner—not in-person—group component	The relationship between the client and peers	Group cohesion (1.1.) and connection (1.3.)	
	Participation setting	No commute time (2.1.)	

^a^The mechanisms that are presented are the summarized mechanisms from Table 2, along with the numbering of the original mechanisms as indicated in Table 2.

### Practitioner—In-Person—Individual

Participants confirmed that (1) *paying attention to drinking behavior*, for example, receiving information about alcohol and health helped them reduce their alcohol use because it created awareness of consequences and made them think about their own use. They also emphasized the importance of specific (2) *communication approaches*, for example, receiving personalized content and conversations based on individual preferences and needs.

Instead of just checking off their checklist, it's about the practitioner sitting there, putting themselves in the other person’s shoes, and asking, “What do you need? What are you asking for? What would you like?” And if you can make that connection, you're halfway there […] and I would create a program with options: “Okay, is there a need for more contact? Can we do something about that?” [RC11]

The participants confirmed that (3) *the relationship between the client and practitioner*, for example, critical and confronting behavior, contributed to reductions in alcohol use because it triggered insights and realization of problems and the need to change. Moreover, trust, a good connection, and not being judged were important aspects in this relationship.

There must be a connection between a practitioner and someone with problematic alcohol use. If there is no connection, it works against the process. [RC2]

In addition, the participants mentioned they preferred a (4) *participation setting* that was anonymous for their environment. Finally, they mentioned that (5)* additional activities*, that is, participating in self-help groups, could help reduce their alcohol use.

### Practitioner—Not In-Person—Individual

Participants mentioned that (1) *paying attention to drinking behavior* was important, for example, by experiencing abstinence and health benefits, because it motivated them to stop consuming alcohol.

You start feeling much better and I could remember nine numbers in a row again. Your memory gets much better, you feel much lighter. When you don’t drink, you think that a drink will relieve the stress and worries you have, but that’s absolutely not true; it’s quite the opposite. [RC19]

Participants identified receiving feedback and reflecting with a practitioner as one of the several important (2) *communication approaches* to reduce their alcohol use. Participants found it particularly important in (3) *the relationship between the client and practitioner* with whom they had frequent and flexible, to stop their alcohol use.

I think that every individual person has their own drinking pattern, with specific times when the cravings are at their strongest. And you should be able to customize interventions for those moments. For example, if you were used to rewarding yourself with a drink around 5:00 PM, you should schedule a call at that time. [RC20]

Also, (4) *involving a relative in treatment* was important, for example, when the relative writes an “impact letter” because it confronted them with the consequences of their use. Finally, participants mentioned that they preferred a (5) *participation setting* that was online because then there was no commute time.

### Practitioner—In-Person—Relatives

Participants confirmed that a partner was more understanding and supportive of them when (1) *the partner was taught to deal with the drinking behavior of the client*. Other working elements in this context were (2) *teaching the partner to approach the client positively*, (3) *providing information about interventions to a partner*, (4) *collaboration between practitioner and relatives,* and (5) *support of relatives*.

It is very important, first of all, that the partner understands the treatment, and second, that the partner can provide support and is also there for the client. And certainly, the client should also pay attention to the partner […] because then you’re walking down that path together. I’ve seen someone who is in [name intervention] with me, and he keeps relapsing, but his partner wants nothing to do with the therapy […]. They are walking next to each other with a big wall in between. [RC8]

### Practitioner—In-Person—Group Component

Participants mentioned that (1) *paying attention to drinking behavior* was an important working element, for example, when they learned how to manage their cravings and drinking behavior, or when the risks of drinking were pointed out. They confirmed that (2) *paying attention to lifestyle*, for example, by motivating lifestyle change helped them reduce their alcohol use.

Alcohol is a part of your lifestyle; why would you address it on its own? Things like snacking, smoking perhaps, other substance use—all of these things should certainly be more strongly motivated […] and hearing the experiences of others and, at some point, thinking, “Yes, that’s one that suits me as well. I’ll try that, too.” [RC3]

In addition, they also mentioned several important (3) *communication approaches*, including receiving feedback, personalized content, and conversations. Participants found it particularly important in (4) *the relationship between the client and practitioner* that the practitioner was a peer support worker because their experiences made the participant feel understood.

They understand the temptations, and they can immediately tell when someone feels guilty when they had a drink, or something went wrong. When someone tries to hide it, they sense it right away. And they don’t judge it because they know it can happen. [RC14]

Participants also mentioned that in (5) *the relationship between client and peers*, the presence of peers with similar levels of (problematic) alcohol use was important. Sharing choices, experiences, struggles, and emotions related to alcohol use and abstinence were also working elements in this relationship because these features helped them learn from each other. Again, they mentioned that a (6) *participation setting* that was anonymous was important. Finally, (7) *additional contact and activities*, for example, individual contact, were helpful for the participants because they provided them more personal attention.

### No Practitioner—Not In-Person—Individual

The participants mentioned that (1) *paying attention to drinking behavior* helped to reduce their alcohol use, for example, with a daily abstinence goal, because “small goals” made abstinence more manageable, and it enhanced a motivational mindset. In addition, in a (2) *participation setting*, for example, online participation possibilities, or location and time-independent participation were helpful because it made participation low threshold. Finally, (3)* additional contact*, for instance, with peers and relatives was mentioned as a working element in this individual context.

So, when you want to use or when you feel like you’ve had enough, they (clients) should be able to call someone. It can also be anonymous, or they can call someone they thrust who can take them out of that situation at that moment. Let’s say regarding a friend who is about to use, or vice versa, he calls me. I listen to him for a while and encourage him. Nine out of 10 times, they won’t use then. [RC11]

### No Practitioner—Not In-Person—Group Component

Participants mentioned that (1) *paying attention to drinking behavior*, for example, by focusing on abstinence, contributed to a reduction in alcohol use. Furthermore, in this context, the presence of someone leading the group was important for (2) *the relationship between the client and peers* for some participants.

In [name group], they don’t want an official leader, but there still needs to be someone who effectively manages things within a group because, in my experience, a group, especially with people with addictions, things can easily spiral into excesses. […] There needs to be a sort of facilitator who is accepted by everyone, or else it won’t work. Otherwise, you get one discussion after another, and it becomes a complicated situation. [RC11]

In addition, sharing experiences and tips with peers motivated them to quit drinking. Finally, (3) *additional contact*, for example, with a practitioner, was helpful because it could help target problems more decisively.

### No Practitioner—In-Person—Group Component

Participants mentioned that (1) *paying attention to drinking behavior*, for example, by bringing attention to or acknowledging one’s problematic use, and (2) *paying attention to lifestyle*, for example, sharing tips for activity engagement, or engaging in them together was important. Moreover, the 12-step Minnesota model was a (3) *communication approach* that helped participants to remain abstinent. Also, sharing experiences was an important element in (4) *the relationship between the client and peers* because it created awareness of the need to change. The participants also mentioned that the (5) *participation setting* played an important role, for example, anonymous participation because it offered security for the participants. *“So, you just feel that sense of safety—you’re simply safe there—and you know what we say here and what we hear here, it stays in this group (…) and because then you can also open up unconditionally.”* [RC10]

Also, (6) *additional activities*, for example, social activities and entering treatment, were important.

I also have to occasionally remind myself that I’m retired now, and I need activities outside the house as well… you see people who just stay at home too much, they get bored, and they become gloomy. Well, sometimes they end up in a downward spiral. And when they hear from me or from others “Well, it’s good to get out, and it can bring you something,” it also helps others. [RC8]

### Practitioner—Not In-Person—Group Component

Participants mentioned that in (1) *the relationship between the client and peers*, starting group treatment with an introduction week was a working element because this could build group cohesion in this relationship. Also, the (2) *participation setting* was important, for example, and online or digital settings, because it eliminated commute time. *“Last year, I even went on vacation with my husband because I participated online with my phone. See, everything could just continue, and the program, too, so it worked out well together.”* [RC19]

## Discussion

### Key Findings

Five elements were found in at least three of the eight contexts and could be labelled as general working elements: (1) *receiving information about alcohol and health (risks)*, (2) *paying attention to abstinence*, (3) *being in contact with, and receiving support of peers and relatives*, (4) *maintaining contact with a practitioner or peer/buddy who was always or flexibly available for communication,* and (5) *learning to manage drinking behavior.* The working elements in specific contexts were *personalized approach*, *reflecting with practitioner*, and *practitioner is peer support worker*, specifically in interventions with practitioner involvement; *anonymous* participation in in-person interventions; *tracking alcohol use* and *the ability to have additional contact with peers* in individual interventions; and *sharing experiences, struggles, and choices* and *peers have similar level of (problematic) alcohol use* were found in interventions in group settings. Moreover, the mechanism *motivation* was found in minimally three contexts.

Our findings were generally in line with the IPT. However, we found several additional working elements and mechanisms not originally included. Based on the confirmed IPT and additions, we created a refined program theory (see Table 2). Below, we discuss the key findings that complemented the IPT.

### Interpretation of Findings

Although “being in contact with others and communicating with them about (alcohol) problems” was identified as a working element in interventions for general adults, but was not identified in the three included studies that focused on interventions for older adults in the review (Boumans et al., 2022), this is the first important addition to the IPT. We expected this, however, because social contact and support from both peers and relatives was important for older adults because of the following aspects.

First, peer contact in interventions had been shown to reduce self-stigma associated with seeking help (Sun et al., 2022). Older adults are notably sensitive to the public stigma associated with alcohol problems (Wadd & Galvani, 2014), and this can be a barrier to seeking treatment for alcohol problems (Keyes et al., 2010). This could explain why older adults in our study emphasized the importance of the sense of recognition and safety provided by peer contact. Social support has been associated with lower levels of public and self-stigma (Birtel et al., 2017; Wallhed Finn et al., 2023).

Second, the importance of contact and support has also been linked to loneliness. Compared to general adults, older adults more often face the loss of relatives and loneliness, which may contribute to negative health effects of alcohol use (Barry & Blow, 2016; Kuerbis & Sacco, 2012). One study with general adults in SUD treatment found that loneliness led to resorting to substance use for connection or coping (Ingram et al., 2020). Additionally, participating in support groups, fostering genuine relationships with others, and engaging in positive, meaningful activities helped alleviate loneliness (Ingram et al., 2020). In our study, without linking these elements to loneliness, the older adults also highlighted that engagement in activities and additional contact with peers and participation in self-help groups during individual interventions helped reduce their alcohol use.

Third, the importance of contact with and support from relatives can be linked to enhanced abstinence. Although older adults in our study had no experience with couple therapy, which helps to reduce alcohol use for adults (Vedel et al., 2008), they acknowledged the importance of involving partners in responding to the CEMO statements. Moreover, they mentioned that relatives can help prevent the resumption of drinking, it fostered motivation, and the clients received better support. These findings show the role that supportive and non-permissive family or social networks can play in minimizing alcohol use (Satre et al., 2004). Future research might focus on perspectives of the relatives of older adults who participate in interventions to examine their willingness to be involved in interventions.

The second important addition to our IPT was that *tracking alcohol use* and *reflecting on use with a practitioner* were important for the older adults. Indeed, older adults have tended to be less aware of their own drinking patterns and associated harm (Rombouts et al., 2023), and they have tended to drink in specific settings, that is, most commonly at homes, restaurants, or at friends’ and acquaintances’ places (Veerbeek et al., 2017) and for specific reasons, for example, social norms and social engagement, habits and daily routines from earlier in life, dealing with bereavement, stress, or anxiety, or the loss of partners, family, or friends (Ward et al., 2011). Tracking and reflecting on their alcohol use could also help provide insights regarding possible erroneous assumptions about someone’s drinking behavior, underlying motives and settings, and these insights could help to focus on someone’s specific needs. For example, drinking in the company of others, for example, with friends, may require different coping skills compared to solitary drinking at home.

The last important addition to the IPT was that motivation was an important mechanism that contributed to a reduction in alcohol use. Motivational interviewing (MI) has helped to enhance the motivation of older adults (Purath et al., 2014). While older adults did not explicitly identify MI as a working element in this study, they did identify working elements intrinsic to this approach, including *empathic behavior of practitioner*, and *not being judged by practitioner*, and *practitioner asks questions and reflects with client*. These findings suggest that MI or MI-related strategies might help foster motivation in interventions aimed at reducing alcohol use among older adults.

Also, in interventions without practitioners, it was important to understand how motivations of older adults are triggered. The older adults in our study derived motivation from both interventions with and without practitioner involvement to abstain through interpersonal interaction (i.e., *contact with peers*) and through *experiencing abstinence (e.g., in a temporary challenge)* and *experiencing health benefits*.

### Limitations

This study had limitations. The first relates to an underrepresentation of identified mechanisms in contrast to all the working elements. Reflecting on and discussing mechanisms require a level of introspection and communication skills which, when combined with the inductive questioning approach, is challenging. The second limitation was that the participations mainly consisted of older adults who were enthusiastic about sharing their experiences. In addition, a portion of the older adult population who were struggling with problematic alcohol use might not be represented in this study because stigma associated with alcohol problems might have led older adults to having difficulties in recognizing the problematic nature of their drinking and avoiding seeking treatment (Hammarlund et al., 2018; Wadd et al., 2011). Third, while we focused on identifying what worked specifically for older adults, we did not compare our findings with other target groups. Consequently, we could not identify which elements were truly age-specific, as the elements and mechanisms identified may also be common for other groups. Additionally, this study relied on what older adults thought was effective, rather than verifying which working elements and mechanisms actually led to abstinence. Future research might investigate which combinations of elements in interventions best contribute to achieving abstinence. For example, conducting a quantitative longitudinal study could assess the effectiveness of elements and mechanisms; abstinence might be measured both immediately after an intervention and over an extended period thereafter.

## Conclusion

In addition to our IPT, which emphasized the importance of providing “information on the consequences of alcohol use” and “personalized feedback about drinking behavior,” our findings emphasized the need for “social contact and support” from both peers and relatives, and “tracking and reflecting on alcohol use” in interventions for older adults, according to the older adults themselves. Furthermore, “motivation” was important for the older adults; motivation could be increased through, for example, the use of MI techniques.

## Supplemental Material

Supplemental Material - Working Elements in Interventions to Reduce Problematic Alcohol use According to Older Adults: A Realist Evaluation Supplemental Material for Working Elements in Interventions to Reduce Problematic Alcohol use According to Older Adults: A Realist Evaluation by Fieke A. E. van den Bulck, Andrea D. Rozema, Dike van de Mheen, Rikste Knijff, Rob H. L. M. Bovens, Sarah E. Stutterheim and Rik Crutzen in Journal of Applied Gerontology.

Supplemental Material - Working Elements in Interventions to Reduce Problematic Alcohol use According to Older Adults: A Realist Evaluation Supplemental Material for Working Elements in Interventions to Reduce Problematic Alcohol use According to Older Adults: A Realist Evaluation by Fieke A. E. van den Bulck, Andrea D. Rozema, Dike van de Mheen, Rikste Knijff, Rob H. L. M. Bovens, Sarah E. Stutterheim and Rik Crutzen in Journal of Applied Gerontology.

Supplemental Material - Working Elements in Interventions to Reduce Problematic Alcohol use According to Older Adults: A Realist Evaluation Supplemental Material for Working Elements in Interventions to Reduce Problematic Alcohol use According to Older Adults: A Realist Evaluation by Fieke A. E. van den Bulck, Andrea D. Rozema, Dike van de Mheen, Rikste Knijff, Rob H. L. M. Bovens, Sarah E. Stutterheim and Rik Crutzen in Journal of Applied Gerontology.
