# Supplementary material for: Working Elements in Interventions to Reduce Problematic Alcohol Use According to Older Adults: A Realist Evaluation
Source: J Appl Gerontol. 2025 Feb 8;44(9):1435–47. doi: 10.1177/07334648241311457 (PMC12335618; doi:10.1177/07334648241311457)
Supplement: Supplemental Material - Working Elements in Interventions to Reduce Problematic Alcohol use According to Older Adults: A Realist Evaluation [file sj-pdf-1-jag-10.1177_07334648241311457.pdf]

Supplementary Material 1: Interviewguide

Background information intervention

- What was your reason for participation in [name intervention]?
- Why this specific intervention?

[DEPENDING ON PRACTITIONER INVOLVEMENT OR NOT]

- What is the role of the professional?
- What does the contact between participant and professional look like?
- What expertise, or qualifications, must a professional have to provide [name intervention]? And why?

Working elements

- What impact do you think [name intervention] has on your actual alcohol consumption?
- What impact do you think [name intervention] has on how your think about alcohol consumption?

- In your opinion, what makes [name of intervention] cause [listed outcomes]?
- What do you think are working elements of the intervention?
  For each element, ask the following things:
  o What works well for whom? Can you explain this?
  o To what extent do you think this works well? Can you explain this?
  o How does this work? Can you explain?
  o Why do you think this works well? Can you explain?

Context

- What is the impact of how [the support/name of intervention] is provided? By provided you can think of it being offered [individually/in a group] and [online/offline]?
  o For whom do you think this mode works well? Can you explain this?
  o To what extent do you think this works well? Can you explain?
  o Why does this work well according to you? Can you explain?
  o Why do you think this works well? Can you explain?
- What is the impact of the location where [the support/name of intervention] is provided?
  o For whom do you think this location works well? Can you explain?
  o To what extent do you think this works well? Can you explain?
  o Why does it works well? Can you explain?
  o Why do you think this works well? Can you explain?

Relatives

- To what extent did you receive support from relatives during [name intervention]?
- o What does this support look like?
- What is the impact of support from relatives? Can you explain?
  o For whom do you think support works well? Can you explain?
  o To what extent do you think this support works well? Can you explain?
  o Why do you think it works well? Can you explain?
  o Why do you think this works well? Can you explain?

- What support would (further) be desirable during participation in [name intervention]? How do you think this could be achieved?

- How do you think relatives experience providing support?
- To what extent do you think relatives need help or support?

CEMO statements

I am going to present a few statements. I would like to invite you to respond to these statements.

Practitioner – in-person – individual (A)

- An intervention with face to face guidance from a professional can make people (55+) reduce or stop drinking.
  - Definitely when there is a focus on drinking behaviour, making people think about their own alcohol use.
  - Definitely when the professional is very empathetic, making participant and professional work together in finding appropriate help and improving their relationship.

Practitioner – not in-person – individual (B)

- An intervention with no face-to-face guidance from a professional can make people (55+) reduce or stop drinking.
  - Definitely when there is personal contact with feedback.
  - Definitely when there is online communication with feedback.

Practitioner – in-person – relatives (C)

- An intervention with face to face guidance from a professional with relatives' involvement can make people (55+) reduce or stop drinking.
  - Defenitely when the partner is taught to deal with the behavior of the drinking partner, this can lead to more understanding and support from the non-drinking partner for the drinking partner.

Practitioner – in-person – group component (D)

- An intervention with face-to-face guidance from a professional in a group setting can make people (55+) reduce or stop drinking.
  - Definitely when people are motivated to make lifestyle changes.
  - Definitely when this is delivered in a workplace setting.

No practitioner – not in-person – individual (E)

- An intervention without guidance from a professional can make people (55+) reduce or stop drinking.
  - Definitely in web based interventions
  - Definitely with telephone based interventions.

No practitioner – not in-person – group component (F)

- An intervention without guidance from a professional in a group setting can make people (55+) reduce or stop drinking.
  - Definitely when the intervention focuses on abstinence, which increases awareness of their alcohol consumption

Closing interview *(5 minutes)*

- Is there anything else you wild like to add to the interview?

- We would like to publicly share the data and information from these interviews and questionnaire you have completed with other researchers. This will allow this information to be reused for new research in the future.
- Before we share this data, we will anonymize all information. This means that personal data or data that can be traced back to you as a person such as name, age or other info that reveals your identity will not be shared with them. Such information will be blacked out before the data is made public.
- Do you give permission to store the anonymized research data for an unlimited period and to make it publicly available to other researchers?
  You are not required to give permission for this. Without permission, we will not make the data publicly available and it will not be used for follow-up research. Even if you do not give permission for this, your data can still be used for current research.

Recorder off

- I would like to thank you for the interview and the time you were willing to spend.
- You will be digitally sent a voucher worth 20 euros.

- Should there be any questions at a later moment, please contact me.
- Also, if you do not have any questions but feel the need to talk after, please contact me.
