# Supplementary material for: Working Elements in Interventions to Reduce Problematic Alcohol Use According to Older Adults: A Realist Evaluation
Source: J Appl Gerontol. 2025 Feb 8;44(9):1435–47. doi: 10.1177/07334648241311457 (PMC12335618; doi:10.1177/07334648241311457)
Supplement: Supplemental Material - Working Elements in Interventions to Reduce Problematic Alcohol use According to Older Adults: A Realist Evaluation [file sj-pdf-2-jag-10.1177_07334648241311457.pdf]

COREQ (COnsolidated criteria for REporting Qualitative research) Checklist

A checklist of items that should be included in reports of qualitative research

Topic	Item No.	Guide Questions/ Description	Author Responses
**Domain 1: Research team and reflexivity**			
Personal Characteristics			
Interviewer/facilitator	1	Which author/s conducted the interview or focus group?	FVDB
Credentials	2	What were the researcher's credentials? E.g. PhD, MD	MSc
Occupation	3	What was their occupation at the time of the study?	PhD
Gender	4	Was the researcher male or female?	Female
Experience and training	5	What experience or training did the researcher have?	Conducted interviews as part of her PhD
Relationship with participants			
Relationship established	6	Was a relationship established prior to study commencement?	Yes
Participant knowledge of the interviewer	7	What did the participants know about the researcher? e.g. personal goals, reasons for doing the research	Created relationship during preparation for the research study. Participants had knowledge of the reasons for doing the research.
Interviewer characteristics	8	What characteristics were reported about the inter-viewer/facilitator? e.g. Bias, assumptions, reasons and interests in the research topic	
**Domain 2: Study design**			
Theoretical framework			
Methodological orientation and Theory	9	What methodological orientation was stated to underpin the study? e.g. grounded theory, discourse analysis, ethnography, phenomenology, content analysis	Interviews with a realist evaluation approach
Sampling	10	How were participants selected? e.g. purposive, convenience, consecutive, snowball	Purposive and convenience sample
Method of approach	11	How were participants	Email or telephone

		approached? e.g. face-to-face, telephone, mail, email	
Sample size	12	How many participants were in the study?	
Non-participation	13	How many people refused to participate or dropped out? Reasons?	18 did not participate. 13 did not respond to our emails or calls, or were not interested in participating. Another five were excluded; one of them due to a failed audio recording and the other four due to their age and exclusion of interventions.
Setting			
Setting of data collection	14	Where was the data collected? e.g. home, clinic, workplace	The interviews with older adults were conducted face to face, online, or by telephone
Presence of non-participants	15	Was anyone else present besides the participants and researchers?	No
Description of sample	16	What are the important characteristics of the sample? e.g. demographic data, date	See Table 1 in manuscript
Data collection			
Interview guide	17	Were questions, prompts, guides provided by the authors? Was it pilot tested?	An interview guide was used, see Supplementary material
Repeat interviews	18	Were repeat interviews carried out? If yes, how many?	No
Audio/visual recording	19	Did the research use audio or visual recording to collect the data?	Audio recordings for Skype and telephone, visual recordings for Microsoft Teams interviews
Field notes	20	Were field notes made during and/or after the interview or focus group?	Yes
Duration	21	What was the duration of the interviews or focus group?	The interviews lasted from 30.7 to 75.1 minutes
Data saturation	22	Was data saturation discussed?	Interviews were conducted until data saturation occurred
Transcripts returned	23	Were transcripts returned to participants for comment and/or corrections?	No
**Domain 3: analysis and findings**			
Data analysis			
Number of data coders	24	How many data coders coded the data?	Two
Description of the coding tree	25	Did authors provide a	The code tree was based on the initial

		description of the coding tree?	program theory, that encompassed the CEMO configurations
Derivation of themes	26	Were themes identified in advance or derived from the data?	Themes were derived from data
Software	27	What software, if applicable, was used to manage the data?	Atlas.TI
Participant checking	28	Did participants provide feedback on the findings?	No
Reporting			
Quotations presented	29	Were participant quotations presented to illustrate the themes/findings? Was each quotation identified? e.g. participant number	Yes, and each quotation is identified
Data and findings consistent	30	Was there consistency between the data presented and the findings?	Yes
Clarity of major themes	31	Were major themes clearly presented in the findings?	Yes, see Table 3 in manuscript
Clarity of minor themes	32	Is there a description of diverse cases or discussion of minor themes?	Yes, see Table 2 in manuscript

Developed from: Tong A, Sainsbury P, Craig J. Consolidated criteria for reporting qualitative research (COREQ): a 32-item checklist for interviews and focus groups. *International Journal for Quality in Health Care*. 2007. Volume 19, Number 6: pp. 349 – 357

Additional Guidelines for Completing the COREQ Checklist for *Journal of Applied Gerontology*:

- This checklist will be published online as supplementary material and we require it to be in the form of a publishable table. Please make sure that material does not bleed outside of cells, etc.
- This checklist is designed to direct readers to relevant material in the manuscript. Where applicable, please direct readers to various sections of the manuscript, such as a Methods section, Conceptual Framework, table or figure. Pages may shift during the publication process so please avoid directing readers to specific page numbers.
- This checklist also is designed to supplement information that may not be reported in the text and/or provide additional details related to information that is reported in the text.

Once you have completed this checklist, please save a copy and upload an anonymized version of it as part of your *Journal of Applied Gerontology* submission. DO NOT include this checklist as part of the main manuscript document. It must be uploaded as a separate supplemental file. If the paper is

accepted, a non-anonymized version should be provided with the final submission of the main manuscript.
