# Supplementary material for: Working Elements in Interventions to Reduce Problematic Alcohol Use According to Older Adults: A Realist Evaluation
Source: J Appl Gerontol. 2025 Feb 8;44(9):1435–47. doi: 10.1177/07334648241311457 (PMC12335618; doi:10.1177/07334648241311457)
Supplement: Supplemental Material - Working Elements in Interventions to Reduce Problematic Alcohol use According to Older Adults: A Realist Evaluation [file sj-pdf-3-jag-10.1177_07334648241311457.pdf]

Supplementary Table 1

Characteristics of included interventions

Intervention**	Contact in intervention with			Setting		Sessions	Target group intervention	
	Professional	Peers	Relatives	Online, telephone	Face to face		Alcohol use***	Older adults (55+ or 65+)
Alcoholics Anonymous		X		X		>1	I	
Apps (Alcohol Freedom, Easyquit, , Selfhelp Alcohol)				X		N/a	I	
CBT for Substance Use	X	X	X*		X	>1	IV	X
Fresh Onwards	X	X	X		X	>1	IV	X
Moti-55	X		X*		X	>1	II	X
NoThanks	X*			X		≥1*, N/a	III	
Online supportgroup		X		X		1, >1*	I	
Schema Therapy	X	X	X*		X	>1	IV	
Sobercare	X	X*	X	X		>1	IV	
Vitality Days	X	X*	X*		X	1	I	X

Note. * = The involvement of this person in the intervention is optional and according on the client's preference. ** Names of (Dutch) interventions are translated: Alcohol Anonymous (*Anonieme Alcoholisten*), Alcohol Freedom (*Alcoholvrijheid*), EasyQuit, Selfhelp Alcohol (*Zelfhulp alcohol*), CBT for Substance Use (*CGT bij middelengebruik*), Fresh Onwards, (*Fris Verder*), Moti-55, NoThanks (*IkPas*), online support groups for abstinence, Schema Therapy (*Schematherapie*), SoberCare, and Vitality Days (Vitaliteitsdagen) *** I = People with all types of alcohol use patterns; II = People with early-stage problematic alcohol use; III = People with all types of alcohol use patterns, except for (heavy) problematic alcohol use or alcohol addiction; IV = People with problematic alcohol use and alcohol addiction.
